# Attitudes toward healthy nutrition in Germany — results from an online-representative cross-sectional survey

**DOI:** 10.3389/fnut.2024.1480980

**Published:** 2025-01-03

**Authors:** Etienne Hanslian, Julia Katharina Schiele, Michael Jeitler, Andreas Michalsen, Manfred Wischnewsky, Maximilian Andreas Storz, Benno Brinkhaus, Miriam Ortiz, Mike R. Sigl, Rasmus Hoffmann, Judith Lehmann, Daniela A. Koppold, Christian S. Keßler

**Affiliations:** ^1^Institute of Social Medicine, Epidemiology and Health Economics, Charité-Universitätsmedizin Berlin, Corporate Member of Freie Universität Berlin and Humboldt-Universität zu Berlin, Berlin, Germany; ^2^Department of Internal Medicine and Nature-Based Therapies, Immanuel Hospital Berlin, Berlin, Germany; ^3^Charité Competence Center for Traditional and Integrative Medicine, Charité – Universitätsmedizin Berlin, Corporate Member of Freie Universität Berlin, Humboldt-Universität zu Berlin and Berlin Institute of Health, Berlin, Germany; ^4^Department of Mathematics and Computer Science, University Bremen, Bremen, Germany; ^5^Department of Internal Medicine II, Centre for Complementary Medicine, Faculty of Medicine, Medical Center, University of Freiburg, Freiburg, Germany; ^6^Institute for Sociology, Otto-Friedrich-University Bamberg, Bamberg, Germany; ^7^Department for Prevention and Care of Diabetes, Department of Medicine III, Faculty of Medicine Carl Gustav Carus, Technische Universität Dresden, Dresden, Germany

**Keywords:** nutrition, diet, public health, online survey, Germany

## Abstract

**Introduction:**

Nutrition plays a crucial role in current German public health strategies. While sociodemographic differences in nutrition have been extensively studied, recent data specific to Germany remains limited.

**Methods:**

An online-representative cross-sectional survey was conducted in 2022 among German-speaking adults aged 18–75 using a Computer Assisted Web Interview (CAWI) format. The survey addressed personal eating habits, the importance of nutrition, motivations behind food choices, and openness to dietary changes. Data analysis included both descriptive and inferential statistics, incorporating CHAID decision tree analysis and nonparametric methods for inductive statistical evaluation.

**Results:**

Among the 4,065 participants, 62.3% regarded healthy nutrition as important, with women, individuals with higher education, and those aged under 26 or over 70 demonstrating greater emphasis on nutrition. CHAID analysis identified education as the most significant predictor of attitudes toward healthy nutrition, followed by sex and income. Participants with a positive attitude toward healthy nutrition reported higher HRQoL scores (EQ-Index = 0.862) compared to neutral or negative attitudes (EQ-Index = 0.835)0.10.5% of participants reported not to eat meat, 28.6% were self-reported flexitarians and 54.1% of participants considered themselves omnivorous. Gender-specific dietary preferences were observed, with plant-based diets being much more popular among females. Participants identified taste preference as the primary factor influencing food choices (77.2%) when asked about the main reasons for their eating habits. Approximately 18% of participants predominantly or exclusively bought organic foods. Interestingly, one third of participants indicated a willingness to adopt a more plant-based diet if recommended by physicians or scientists.

**Conclusion:**

This online representative survey revealed significant associations between nutrition and sociodemographic aspects. Understanding this complex interplay might be useful for public health nutrition strategies that promote healthier national eating patterns.

## 1 Introduction

Public health strategies in Germany are increasingly recognizing the importance of nutrition, but recent data on sociodemographic differences in nutrition remain limited. The factors influencing food choices are complex and multilayered, ranging from taste preferences to ethical considerations (1). Food choices are also driven by a growing public awareness of the environmental and health implications of food choices. The 2023 German Federal Ministry of Food and Agriculture (German: Bundesministerium für Ernährung und Landwirtschaft: BMEL) Nutrition Report indicates evolving consumer preferences toward healthier, more sustainable, and ethically produced food, particularly with regard to animal products (2). The report highlighted a significant decline in daily meat consumption from 34% in 2015 to 20% in 2023, coupled with an increased consumption of plant-based foods. Ten percent of participants refrained from consuming meat at all, with younger people increasingly adopting plant-based diets, and 46% of participants consciously reducing their meat consumption, categorized as flexitarians (3).

Plant-based dietary patterns recently gained increasing attention for their numerous environmental and health benefits, particularly in comparison to the highly prevalent (omnivorous) Western diet (4, 5). A systematic review analyzing 141 studies compared nutrient intake and health data of adults following plant-based (vegetarian and vegan) diets with those consuming meat on a regular basis. Plant-based diets exerted greater benefits with regard to the intake of several nutrients of public health concern (e.g., fiber), emphasizing the need for public health strategies promoting a regular intake of nutrient-dense plant-based foods (6).

In response to global health and environmental challenges and with regard to the rising prevalence diet-related non-communicable diseases, the German Federal Government initiated the “Food and Nutrition Strategy” following the Second International Conference on Nutrition (ISCN2) (7). This strategy is designed to path the way for a tectonic shift toward plant-based diets and toward reducing the consumption of ultra-processed products high in fat, processed sugars, and salt. The strategy was also designed to address the health challenges linked to a poor nutrition, including overweight, obesity, and other highly prevalent lifestyle-related diseases in Germany. The overarching objective is a broader transformation of the food system, toward a more sustainable framework (8). Additionally the BMEL published a nutrition strategy paper highlighting public health nutrition strategies such as an improvement of communal catering, the promotion of plant-based diets and to secure access to sustainably and ecologically produced food (9).

This strategy is in line with the IN FORM initiative, a German national action plan initiated in 2008 to combat poor nutrition, lack of physical activity, overweight, and lifestyle related diseases. IN FORM has implemented over 200 projects aimed at improving sustainable eating and exercise habits in Germany. This initiative places a strong emphasis on nutritional education and the promotion of nutritional expertise across all age groups (10). Similarly, the COPLANT (COhort on PLANT-based Diets) study, launched at the Federal Institute for Risk Assessment (German: Bundesinstitut für Risikobewertung) and associated centers, targets a comprehensive assessment of plant-based diets, evaluating their health implications with the goal of developing evidence-based nutritional guidelines to support public health and sustainable eating habits (11).

Moreover, the proposal to restructure the value-added tax (VAT) system in Germany, as suggested by the German Environment Agency, includes a critical analysis of current tax practices. For instance, the reduced VAT on animal products is identified as an environmentally detrimental subsidy. The existing VAT system in Germany is criticized for its fragmented regulations that often overlook ecological concerns and largely neglect social issues. The proposed VAT realignment seeks to address these shortcomings by promoting environmentally friendly products and fostering a more cohesive, environmentally conscious tax framework (12). Adjusting prices through taxes could be a potential lever to encourage greater consumption of vegetarian meals (13–15). Additionally, providing more vegetarian options could effectively reduce the selection and purchase of meat (16).

Plant-based diets offer dual benefits by positively impacting both personal health and environmental sustainability known as planetary health (4, 17, 18). Plant-based diets have gained recognition for their potential health benefits, particularly in reducing the risk of cardiovascular diseases (17). The EAT-Lancet Commission has developed a strategy for agriculture and nutrition aimed at safeguarding both human and planetary health. To stay within the planet’s boundaries, the consumption of vegetables, fruits, legumes, and nuts must at least double by 2050, whereas meat and sugar consumption must be reduced by at least 50% in the same time frame. Alongside changes in diet, improvements in food production and significant reductions in food waste are necessary. The aim was to establish a scientific basis for transforming the global food system, resulting in the “Planetary Health Diet” (PHD) (4). By advocating for a diet that is not only healthy for individuals but also sustainable for the planet, the PHD encapsulates the essence of the Sustainable Development Goals (SDGs) (especially SDG 3), which is dedicated to healthy lives and well-being for all at all ages (4, 18).

Flexitarian diets, emphasizing plant-based foods while allowing occasional consumption of animal products, are considered more approachable and sustainable for individuals transitioning from a traditional Western diet compared to strictly vegan or vegetarian diets, being nutritionally adequate (19, 20). These diets not only provide nutritional adequacy but also contribute to environmental sustainability by reducing meat consumption and potentially decreasing food waste (4). The flexibility of flexitarian diets may encourage a broader segment of the population to adopt more plant-based eating habits, thereby amplifying the positive impacts on health and the environment (21).

Dietary preferences and the adoption of plant-based diets are significantly influenced by various sociodemographic factors, including sex, age, education, and cultural background (22). Younger generations in particular, are showing a growing interest in plant-based diets, often motivated by environmental concerns and animal welfare considerations. However, they may also prioritize convenience foods due to lifestyle factors, which can sometimes conflict with the pursuit of healthier food choices (23). Understanding these factors is crucial for developing targeted strategies to promote plant-based diets across different population groups.

While global trends indicate an increasing shift toward plant-based diets, there is limited up-to-date evidence on how these sociodemographic factors specifically influence nutrition in Germany (23). The aim of this survey was to gain a more comprehensive understanding of the prevailing attitudes toward healthy nutrition in Germany, focusing on the influence of sociodemographic variables on food choices.

## 2 Methods

The present study utilizes data from a cross-sectional survey carried out from September to October 2022 by the Institute of Social Medicine, Epidemiology and Health Economics of Charité - Universitätsmedizin Berlin about the use and acceptance of TCIM (Traditional, Complementary and Integrative Medicine) (24). The survey was conducted online using the software “Computer Assisted Web Interview” (CAWI) with German-speaking residents aged between 18 and 75. A detailed description of the design of the main study has been published previously (24). Data collection, processing, and storage were performed in accordance with international guidelines for good clinical practice (Declaration of Helsinki, ICH-GCP) and research ethics for accompanying sociological research (25). The Charité Ethics Committee approved this study, which was registered at ClinicalTrials.gov (NCT05530720). In compliance with the international standard ISO 26362, the online panel’s sampling quality was both monitored and certified. The main study was a comprehensive approach combining a representative cross-sectional survey with a qualitative component. Questions included socio-demographic data of the participants, TCIM utilization, attitudes toward TCIM, types of applications, background knowledge on TCIM, the role of TCIM within the Covid-19 pandemic context, as well as questions on nutrition, Ayurveda, the Sinus Milieu® Indicator, and the EQ-5D-5L questionnaire on quality of life. Findings related to domains other than nutrition can be found in separate publications (24, 26).

The questionnaire included eight nutrition-related questions. Questions inquired about dietary habits, the subjective importance of nutrition, the consumption of certified organic or sustainable products, and about potential dietary changes subsequent to recommendations from physicians or scientists (Supplementary Table S1).

The selection of participants was quota-based and representative of the various age groups, genders, educational backgrounds, and geographic regions of Germany. The specifications for these quota were aligned with the procedures used in the best4planning (B4P) study, which is recognized for its ability of generating representative samples (27). For the original B4P study, over 30,000 individuals were randomly chosen from the population in Germany. To ensure the sample mirrored the demographic composition of the general population, quota controls were applied to correct any socio-demographic imbalances.

### 2.1 Sinus-milieus®

Sinus-Milieus® (28) is a widely used sociological tool that categorizes individuals into groups of like-minded people based on shared values, lifestyles, and similar social circumstances. Developed by the SINUS-Institute, this model has been used in the German-speaking market for decades and is now available in over 48 countries. Key aspects of Sinus-Milieus are summarized below (14):

1. Grouping by lifestyles: Sinus-Milieus summarize people according to their lifestyles, creating distinct clusters. These clusters represent different segments of society, each with its own set of values, goals, and communication preferences.
2. Permeable boundaries: The boundaries between these groups are fluid. People can move between milieus based on changes in their circumstances, experiences, or values.
3. Social status and value orientations: Sinus-Milieus cover a spectrum of social statuses, ranging from lower to higher. Additionally, they reflect a range of value orientations, from traditional to postmodern.
4. Application in research and marketing: Researchers, marketers, and policymakers use Sinus-Milieus to gain insights into societal trends, consumer behavior, and communication strategies. By understanding these distinct groups, they can tailor their approaches effectively.
5. Outcome variables: In our specific study, Sinus-Milieus were used to explore the perceived importance of healthy nutrition.

### 2.2 EQ-5D-5L index for health-related quality of life

The EQ-5D-5L questionnaire developed by the EuroQol Group (29–31) is a widely used generic instrument to measure health-related quality of life (HRQoL) consisting of two parts. The first part (the descriptive system) assesses HRQoL across five dimensions (mobility, self-care, usual activities, pain/discomfort, and anxiety/depression), each of which has five levels of response (no problems, slight problems, moderate problems, severe problems, extreme problems/unable to). A respondent’s health state is represented by a five-digit profile, where each digit corresponds to a severity level [1 (no problems) to 5 (extreme problems)] on one of the five dimensions. Each health state can potentially be assigned a specific health state index score based on societal preference weights for the health state levels. Health state index scores generally range from less than 0 (an index value of <0 represents a hypothetical health state worse than death, where 0 is the value of a health state equivalent to dead) to 1 (perfect health), with higher scores indicating higher health utility. These 25 weights, one for each health state level, are determined for each country based on population-representative data. The EQ-5D Index (EQ-Index) of a patient is the sum of the corresponding weights.

### 2.3 Statistical analysis

For continuous variables, we report means and standard deviations if normally distributed, and medians and interquartile ranges otherwise. Categorical variables are presented as frequencies and percentages. Normality was assessed using the Kolmogorov–Smirnov test. Differences in categorical variables were analyzed with the Pearson’s *χ*^2^ test or Fisher’s exact test. Crosstab analysis revealed patterns, correlations, and trends among categorical variables. The Kruskal-Wallis test was used to compare non-normally distributed continuous variable across more than two groups. Dunn’s test was employed as a post-hoc test for Kruskal-Wallis. When additional covariates and factors were included in the model with non normally distributed continuous variables we used Quade’s nonparametric ANCOVA as a specialized General Linear Model. An alternative approach would be “aligned ranks transformation ANOVA” (ART ANOVA), which allows multiple independent variables, interactions, and repeated measures (R-library ARTool).

To obtain Quade’s nonparametric ANCOVA we ranked both the dependent variable (EQ-5D-5) and the covariate (age). A linear regression was then performed, regressing the ranks of the dependent variable on the ranks of the covariates and saving the unstandardized residuals (ignoring the grouping factor). In the final step, an univariate General Linear Model (GLM) was conducted, using the residuals from the previous regression as the dependent variable and the grouping variable as the factor. The F-test resulting from this GLM is the nonparametric Quade test.

Additionally, we used the Exhaustive CHAID (Chi-squared Automatic Interaction Detector) decision tree algorithm to predict a target variable based on the data. All statistical analyses were performed using R (version 4.3) and SPSS (version 29).

## 3 Results

Of the 4,065 survey participants aged 18 to 75, 51.7% were female, 47.9% male, and 0.4% identified as diverse. The mean age was 49.3 years (standard deviation 15.8), the median age was 51.0 years with interquartile range 27. Educational attainment was distributed as follows: 42.5% had higher education (university degree or equivalent) and 56.8% had primary or secondary education. Among participants, almost 70% reported using TCIM at some point in their lives, with 32% using it within the past 12 months and 18% currently using it. For detailed sociodemographic characteristics, please refer to the main publication (24). Among the 1,291 participants (31.8%) who used TCIM within the past year, women were overrepresented (60.7%). Approximately half (54.3%) reported a net household income between €2,000 and €5,000, with 7.5% earning above this range and 38.2% earning below it. Table 1 displays the basic characteristics and importance of healthy nutrition.

**Table 1:** Basic characteristics and importance of healthy nutrition.

Category	Subcategories	*n*	%	“Healthy nutrition is an important topic in my life”			
				Very important/important (%)	Neutral or I do not know (%)	Less important/completely unimportant (%)	*p*-value
Gender	Male	1947	47.9	57	32.8	10.3	<0.001
	Female	2,101	51.7	67.4	25.2	7.4	
	Diverse	17	0.4	52.9	41.2	5.9	
Age in years	Under 20	67	1.6	56.7	32.8	10.4	0.016
	20 to 29	557	13.7	65	27.8	7.2	
	30 to 39	631	15.5	59.6	30.3	10.1	
	40 to 49	656	16.1	61	30.6	8.4	
	50 to 59	896	22	58.8	30.4	10.8	
	60 to 75	1,258	66.1	66.1	26.5	7.4	
Educational level	No general school-leaving certificate (yet)	30	0.7	46.7	36.7	16.7	<0.001
	Secondary (elementary, basic)	251	6.2	40.2	43.8	15.9	
	Secondary school with apprenticeship/vocational training	885	21.8	55	35.4	9.6	
	Secondary school without A-levels	1,173	28.9	60.2	30.6	9.2	
	A-levels, (technical) university entrance	751	18.5	67.5	26	6.5	
	Studies (university, college, etc.)	949	23.3	73.4	19.3	7.3	
	PhD	26	0.6	88.5	11.5	0	
Net monthly household	Up to 1,000 €	505	12.4	51.3	36.8	11.9	<0.001
	1,000–2000 €	1,047	25.8	58.6	30.4	11	
	2000–3,000 €	1,049	25.8	62.4	30.5	7.1	
	3,000–4,000 €	735	18.1	65.7	26	8.3	
	4,000–5,000 €	424	10.4	71.5	23.1	5.4	
	>5,000 €	305	7.5	72.5	20	7.5	
Sinus milieus	Conservative upscale milieu	429	10.6	78.3	18.2	3.5	<0.001
	Post-materialist milieu	575	14.1	76.9	19.5	3.7	
	Performer milieu	452	11.1	67	24.1	8.8	
	Expeditive milieu	417	10.3	71.2	21.3	7.4	
	Adaptive-pragmatic middle class milieu	475	11.7	53.9	35.8	10.3	
	Nostalgic middle class milieu	443	10.9	53.5	34.5	12	
	Traditional milieu	289	7.1	56.4	37	6.6	
	Precarious milieu	346	8.5	37.6	43.4	19.1	
	Consumer-hedonistic milieu	314	7.7	46.2	40.1	13.7	
	Neo-ecological milieu	325	8	69.5	24.6	5.8	
Religious community	Catholic	853	21	63.3	28.6	8.1	0.143
	Protestant	1,051	25.9	63.5	29.1	7.4	
	Muslim	69	1.7	73.9	20.3	5.8	
	Buddhist	17	0.4	58.8	35.3	5.9	
	Hindu	2	0.1	0	50	50	
	Jewish	12	0.3	83.3	16.7	0	
	Other	72	1.8	56.9	30.6	12.5	
	No religious affiliation/atheist	1989	48.9	61.1	29.1	9.8	
Location size	Under 2,000 inhabitants	273	6.7	63	30.8	6.2	0.02
	2,000 to under 5,000 inhabitants	232	5.7	57.3	32.3	10.3	
	5,000 to under 20,000 inhabitants	603	14.8	59.4	31.3	9.3	
	20,000 to under 50,000 inhabitants	557	13.7	59.6	31.4	9	
	50,000 to under 100,000 inhabitants	401	9.9	59.9	32.2	8	
	100,000 to under 500,000 inhabitants	889	21.9	62.3	28.2	9.4	
	500,000 inhabitants and more	1,110	27.3	67.2	24.4	8.4	

### 3.1 Importance of healthy nutrition

**Figure 1:**
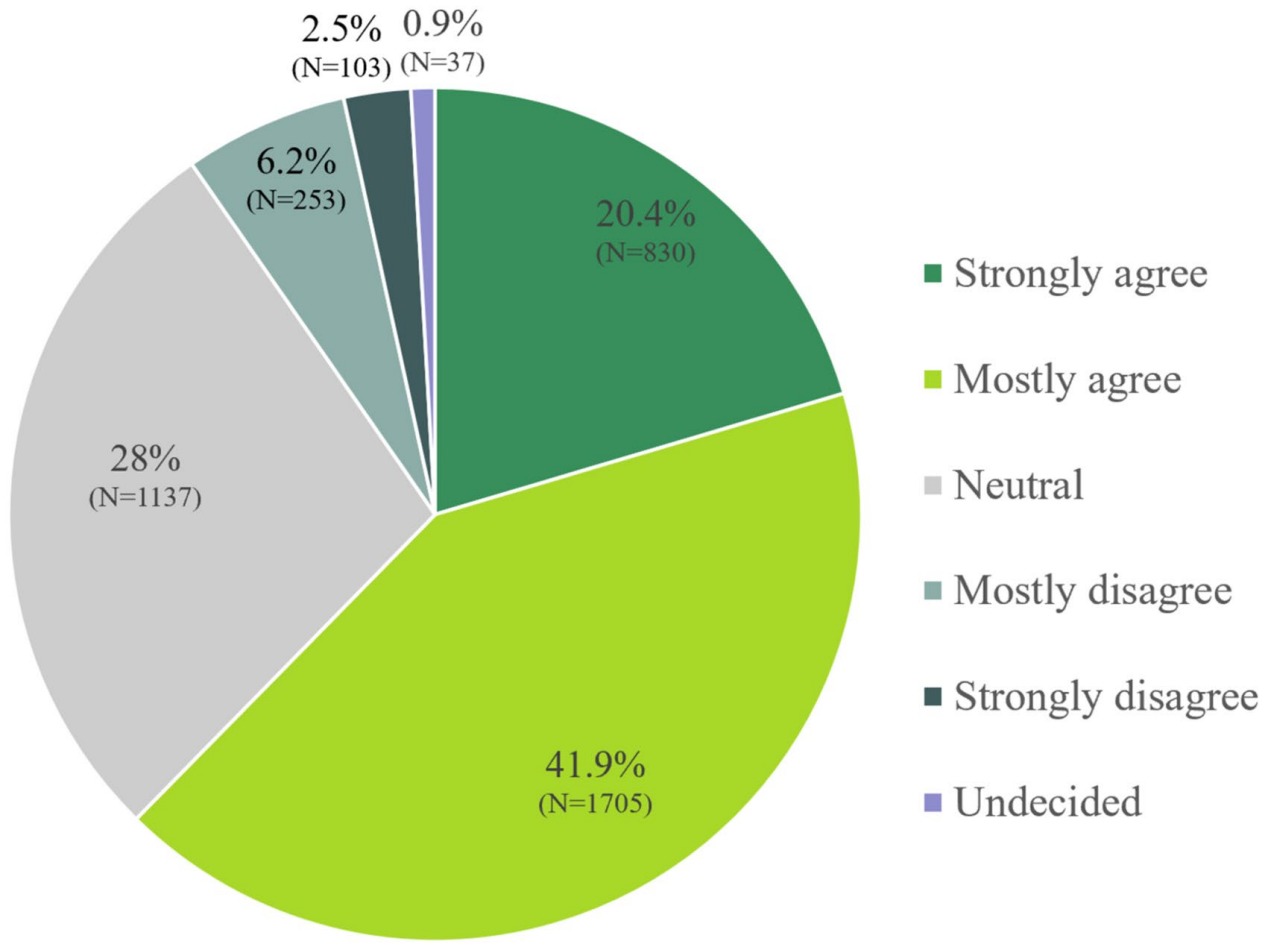
“Healthy nutrition is an important topic in my life”.

The examination of dietary preferences indicated that approximately two-thirds of the sample (62.3%) regard healthy nutrition as either very important (20.4%) or moderately important (41.9%) (Figure 1).

Significant differences between the subjective importance of nutrition and sociodemographic data were observed (Table 1). Significant differences were found for sex, age, education, net monthly household income, Sinus milieu® categorization and size of the place of residence. Female participants tended to place significantly (*p* < 0.001) greater emphasis on nutrition than male participants.

Among the participants, 88.5% with a PhD and 73.4% with an academic degree perceived healthy nutrition as important or very important. In contrast, only 40.2% of individuals with a secondary (elementary, basic) school-leaving qualification and/or without completed apprenticeship/vocational training found healthy nutrition important or very important. Furthermore, 72.5% of households with a monthly net income >5,000€ rated healthy nutrition as important or very important, whereas this applied to 51.3% in the lowest income category (<1,000€; Table 1).

### 3.2 The sinus milieu® model and attitudes toward nutrition

The Sinus Milieu® model is a classic tool for societal analysis. The Sinus-Milieus® arrange people with similar values, a similar lifestyle and a comparable social situation into groups of “like-minded people” (section 2.1).

**Figure 2:**
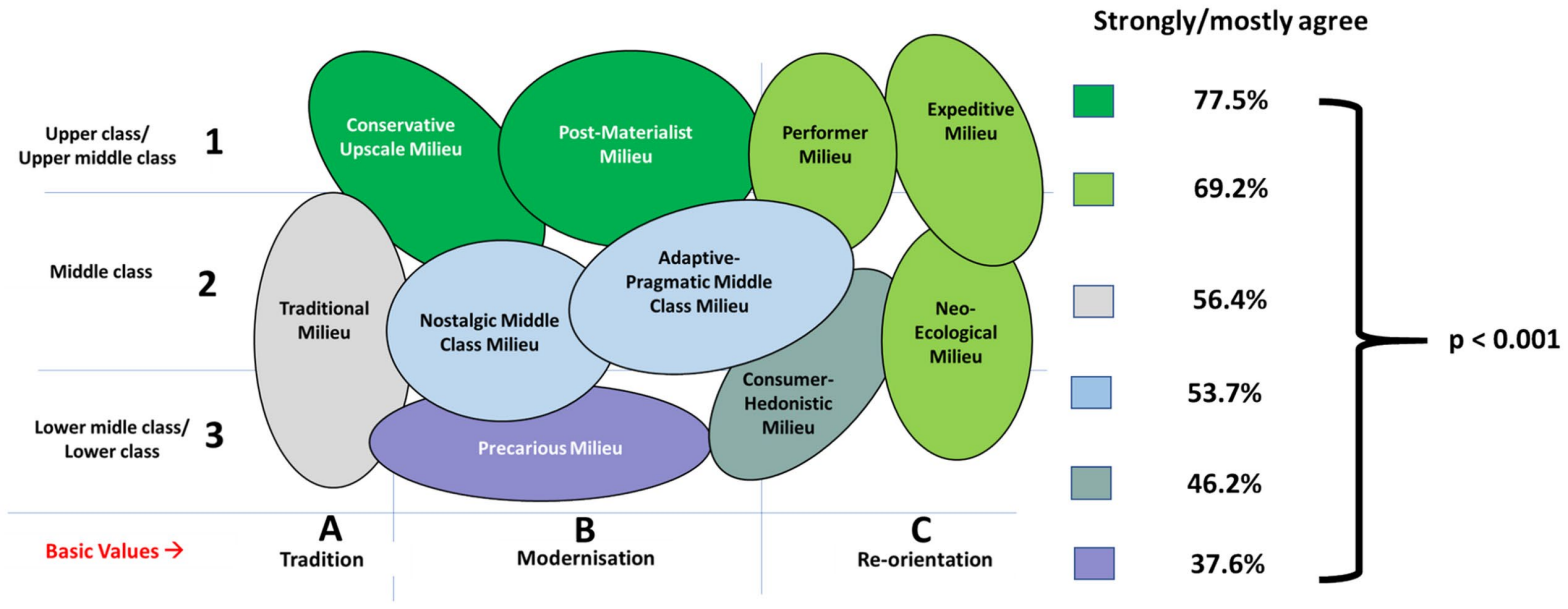
Sinus-Milieus® and percentage of people who find healthy nutrition an important topic in their life.

There were significant (*p* < 0.001) differences between Sinus-Milieus® and attitudes toward nutrition. The Precarious Milieu exhibited the lowest percentage of people (37.6%), who strongly or mostly agree, while the Conservative Upscale Milieu and the Post-Materialist Milieu demonstrated the highest (77.5%). There were no significant differences between milieus in the same color category (Figure 2).

### 3.3 Importance of healthy nutrition

Next, we examined subgroups based on their agreement with the importance of healthy nutrition. Overall, 62.3% of participants viewed healthy nutrition as very or mostly important whereas 8.8% viewed it as very or mostly unimportant. We took the following seven parameters as independent variables: attitude toward TCIM, gender, total income, high-school diploma, age category, health status, and Sinus Main Milieus**®**. The dependent variable is ‘importance of healthy nutrition in one’s life’. The most significant variable in the decision tree model (Supplementary Figures S1A–C) was the attitude toward TCIM, which is divided into the following three categories: (1) strongly or mostly positive, (2) neutral or undecided, and (3) mostly or very negative.

If the attitude toward TCIM was strongly or mostly positive, three subgroups of individuals who considered healthy nutrition to be a very important or mostly important topic of their life emerged (with probabilities exceeding 80%):

- 85.8% if the corresponding Sinus main milieu**®** is ‘Milieus of the Future’ and the individual is over 40 years old (3.9% very unimportant or mostly unimportant).
- 86.6% or 80.9% if the corresponding Sinus main milieu**®** is ‘Society’s Leading Milieus’ and the individual identifies as female or male/diverse, respectively (1.0% or 5.1% very unimportant or mostly unimportant, respectively).

When ‘over 40 years old’ was replaced with ‘between 30 and 39 years old’ in the first subgroup, the percentage of participants who considered nutrition important or mostly important decreased from 85.8 to 69.2% (Supplementary Figure S1A).

When the attitude toward TCIM was neutral or undecided, and the total income was less than €1,000, the overall probability that an individual considered healthy nutrition to be a very important or mostly important topic in their life decreased to 22.2% in the Sinus main milieus**®** Modern Mainstream or Traditional Mainstream (Supplementary Figure S1B).

When the attitude toward TCIM was mostly or very negative, the overall probability that an individual considered healthy nutrition to be a very important or mostly important topic in its life was still 39.1%. This indicates that a mostly or very negative attitude toward TCIM does not necessarily equate to disagreement with the importance of healthy nutrition. The next significant variable in this subgroup with a negative attitude toward TCIM was a high school diploma. 50.9% of participants with a high school diploma considered healthy nutrition important compared to 31.6% without a high school diploma (Supplementary Figure S1C).

Also, an association existed between the importance of nutrition and age (*p* = 0.013), while those over 70 years (70.3%) and those under 26 years old (65.7%) scored highest. The age groups 26 to 60 years (60.0%) and 60 to 70 years (64.1%) still expressed considerable interest (“important/very important”) (Supplementary Figure S2).

### 3.4 Dietary habits

**Figure 3:**
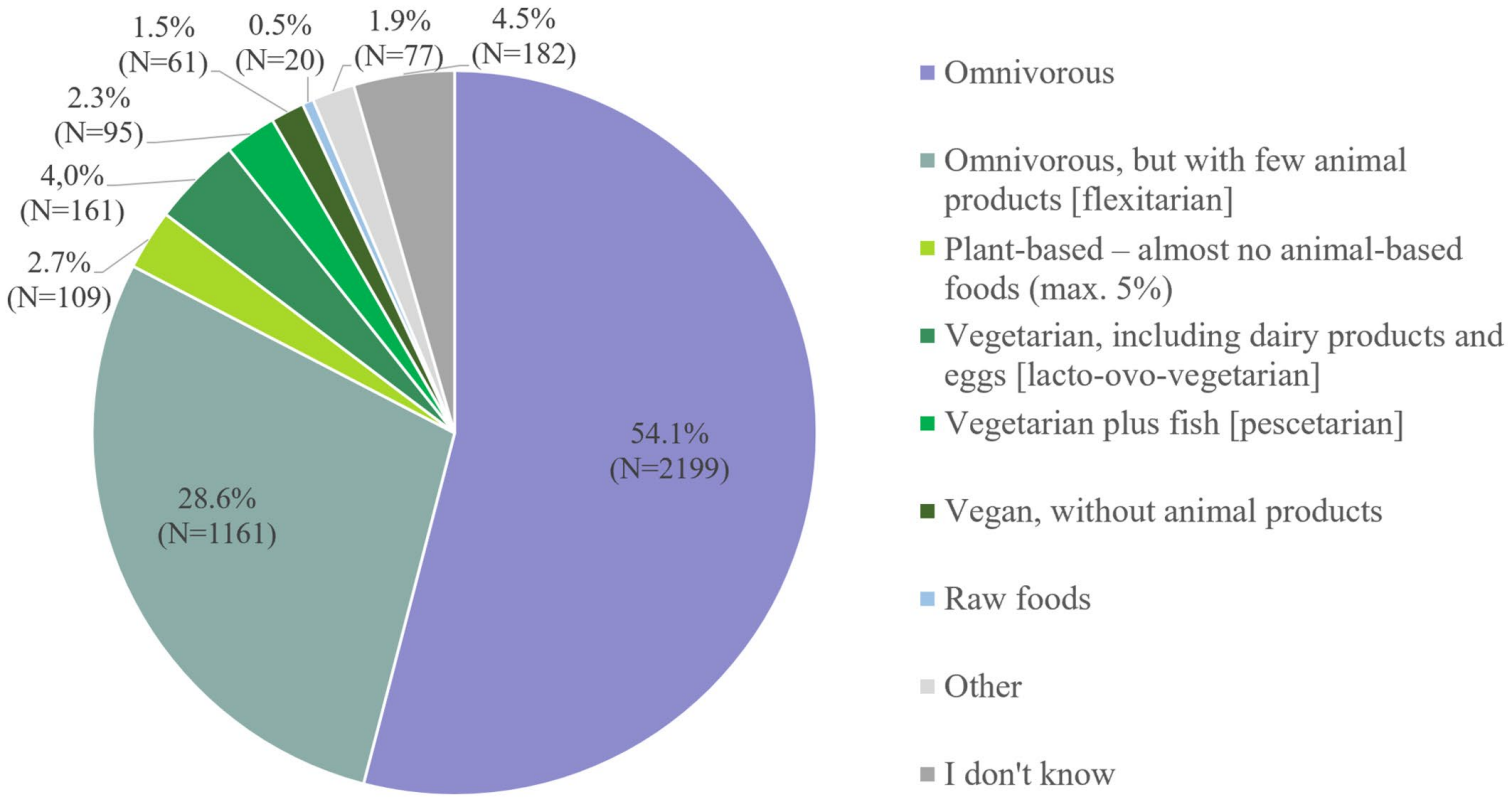
Dietary habits of participants.

Regarding dietary habits, 10.5% reported to refrain from consuming meat. Out of those, 4.0% were self-reported lacto-ovo-vegetarians, 1.5% vegans (Figure 3).

**Figure 4:**
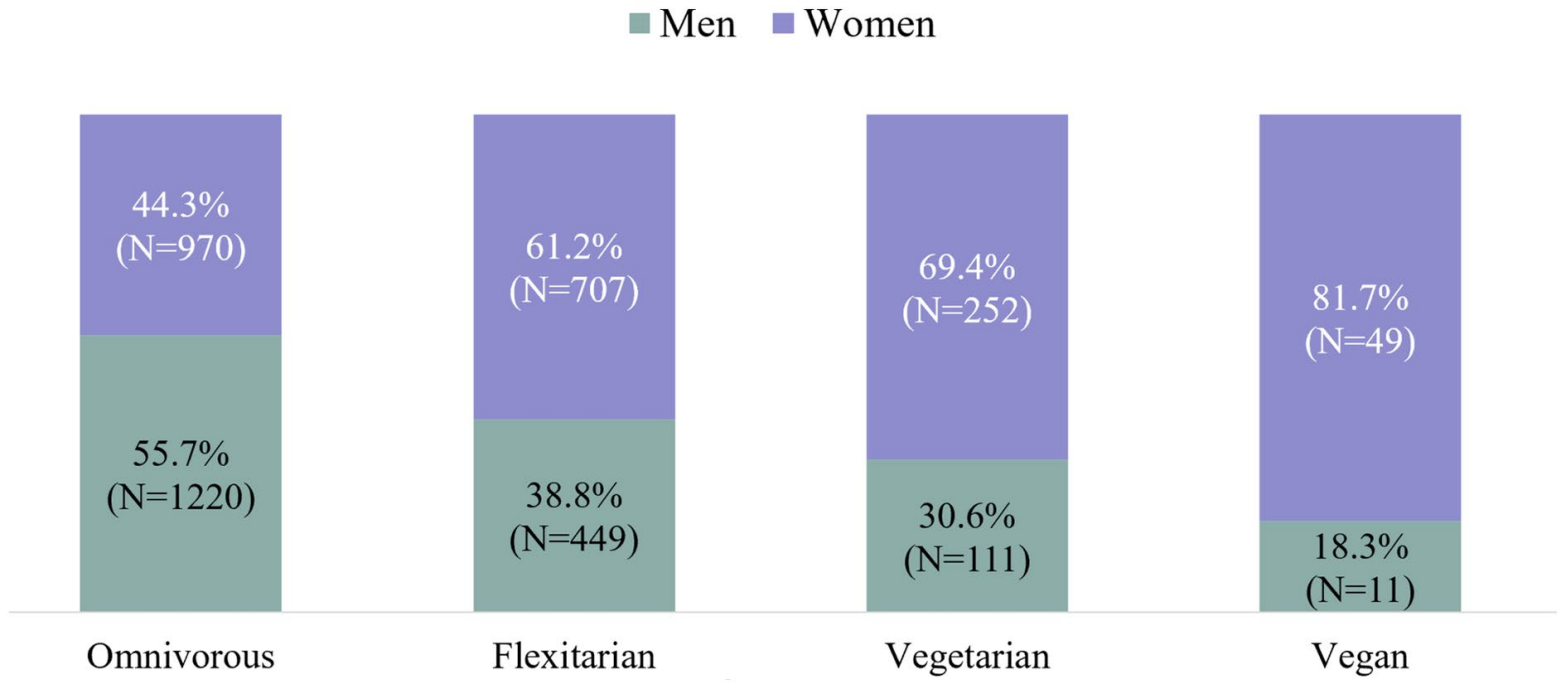
Proportion of women and men among omnivorous, flexitarian, vegetarian and vegan diets – [Legend: Vegetarians consist of: (1) plant-based – almost no animal foods (max. 5%), (2) vegetarian, including dairy products and eggs (lacto-ovo-vegetarian) and (3) vegetarian including fish (pescetarian)].

Subsequently, we examined potential associations between sex and meat intake. Hereby, Figure 4 shows a clear trend: The more meat-based the self-reported dietary pattern, the lower the percentage of females (and vice versa) (Figure 4).

### 3.5 Reasons and influences for food choices and organic-food use

**Figure 5:**
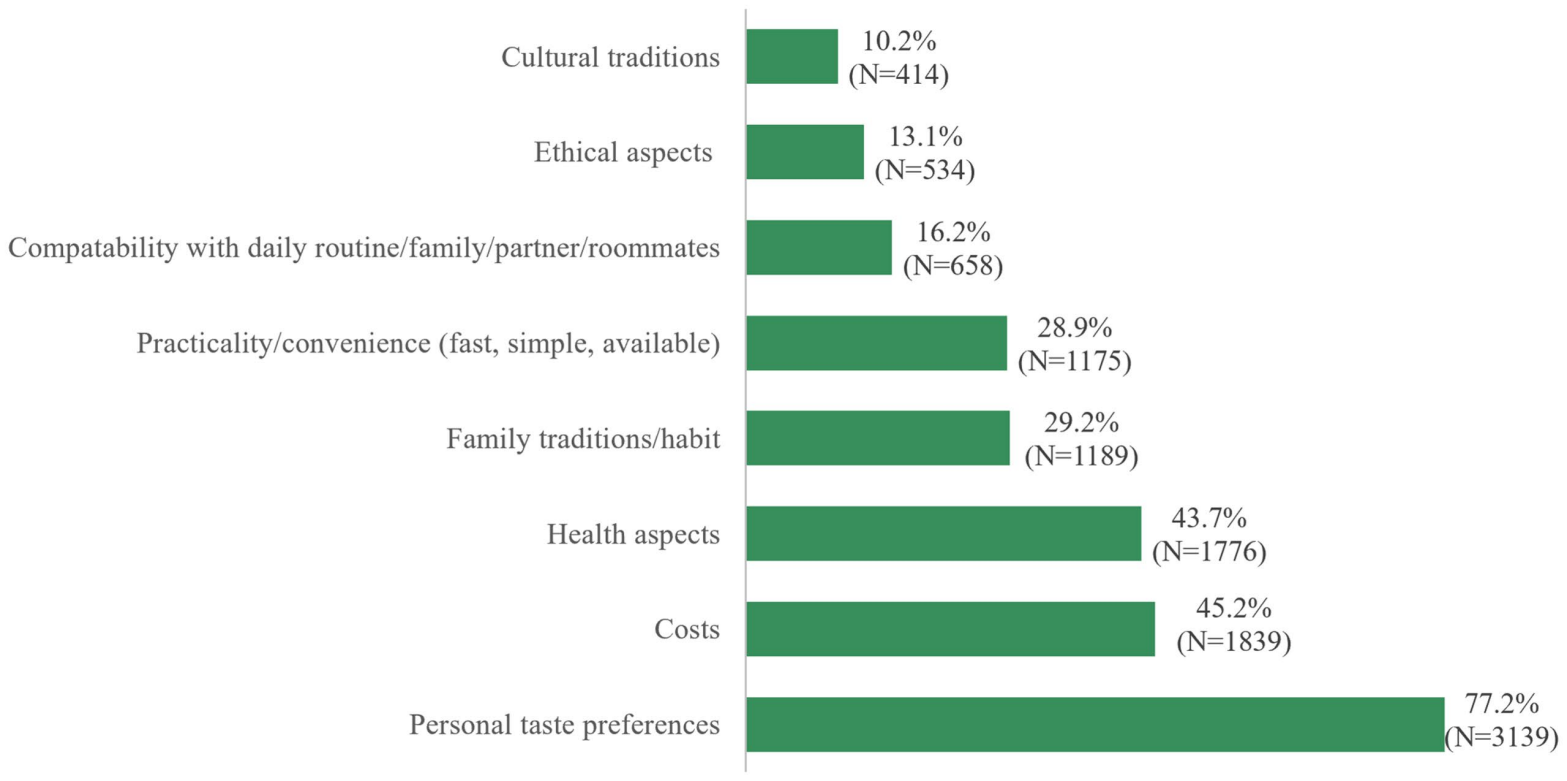
“My main reasons for the way I eat” (maximum 3 mentions, percent of cases).

Over three-quarters of the participants based their food choices on taste preferences (77.2%), followed by cost aspects and health considerations (45.2 and 43.7%, respectively). One third of participants were also influenced by family traditions/habits or practicability (Figure 5).

**Figure 6:**
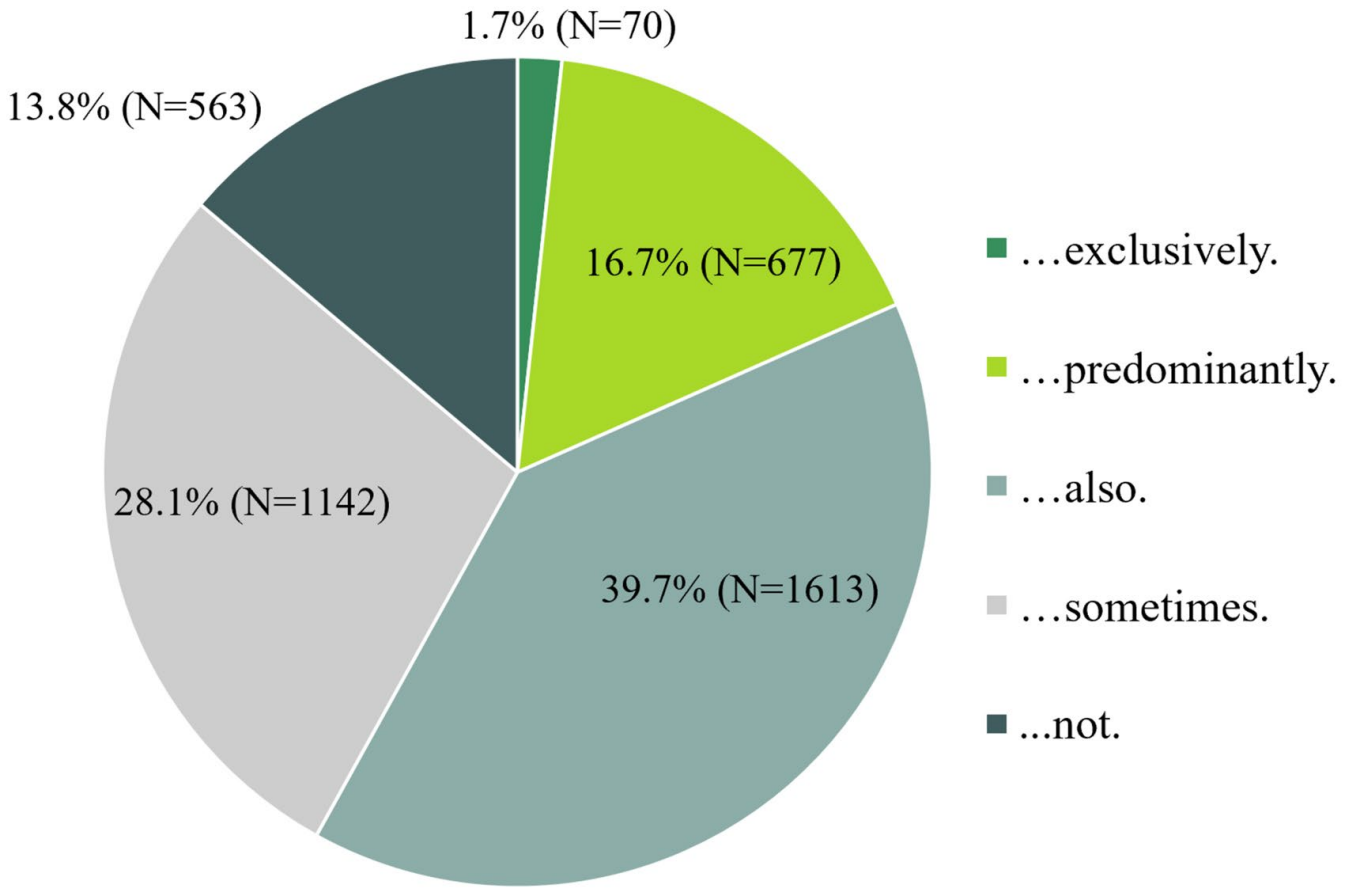
“Which answer option applies to you? I buy organic food…”.

18.4% of participants predominantly or exclusively purchased organic foods, while a substantial proportion (39.7%) chose a combination of conventional and organic foods (Figure 6).

**Figure 7:**
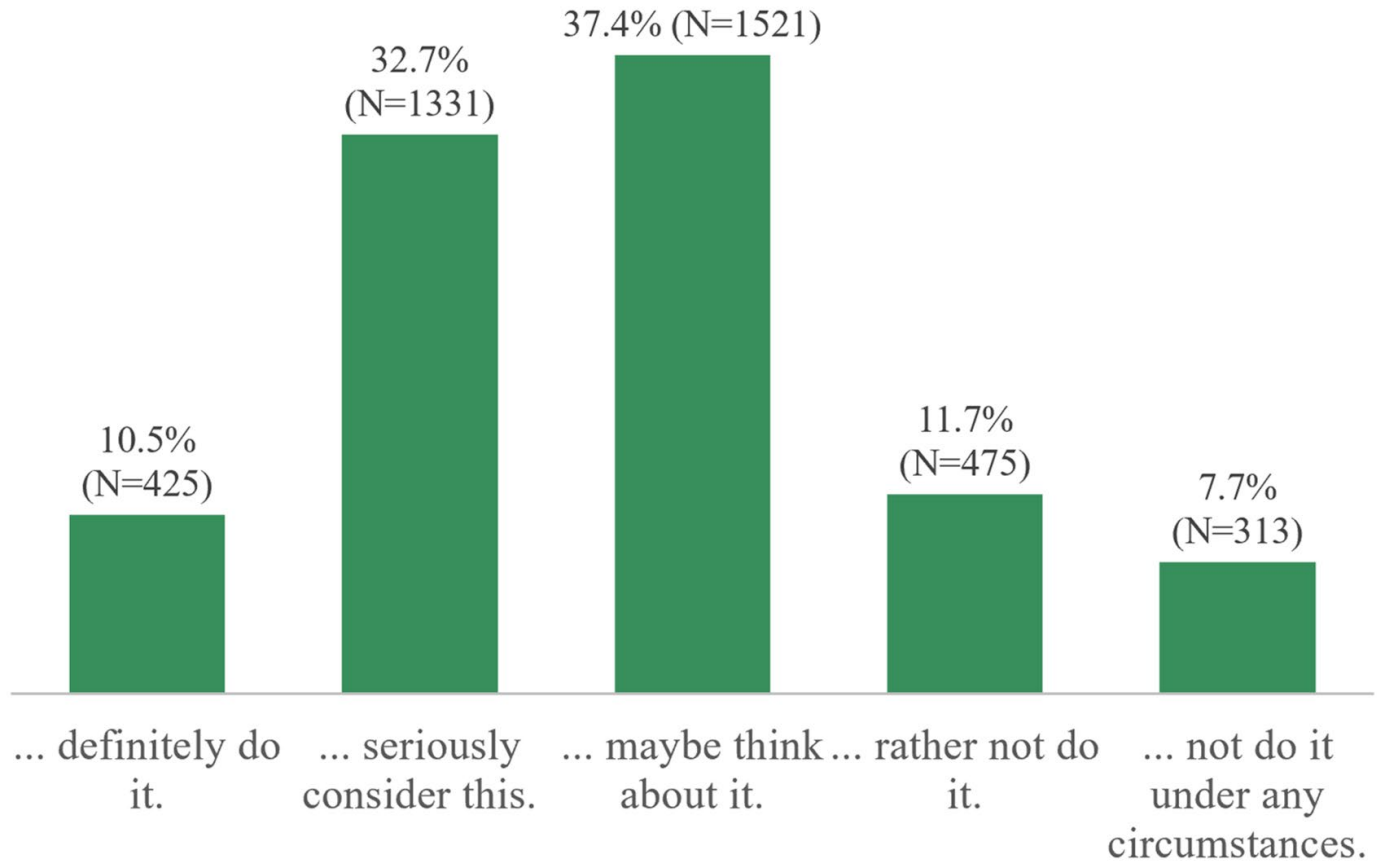
“Which answer option applies to you? If doctors or scientists recommended that I should avoid animal products, I would…” (*n* = 4,065).

If doctors or scientists recommended avoidance of animal products, almost one third of individuals would seriously consider it, while another 37.4% of participants indicated that they would maybe think about it (Figure 7).

**Figure 8:**
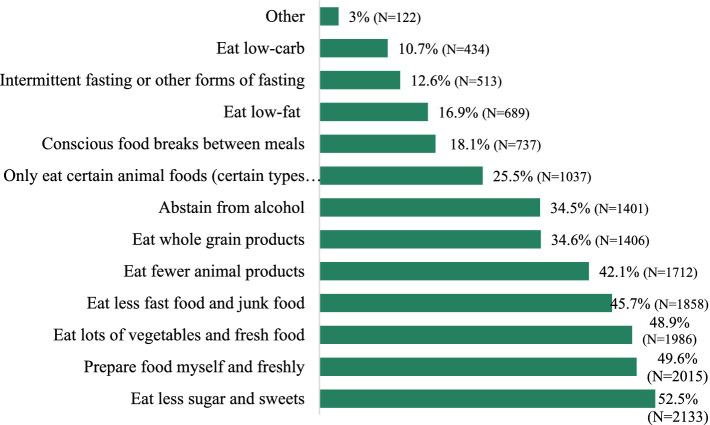
“What are you already consciously doing for health reasons in relation to your diet?” (Multiple answers possible).

In terms of practices for healthy nutrition, approximately 50% of the participants indicated they had already reduced their consumption of foods high in processed sugar (52.5%), prepared fresh meals (49.6%) and consumed higher amounts of fresh vegetables (48.9%). Additionally, many participants reported eating less fast food and junk food (45.7%) or indicated a reduced intake of animal products (42.1%) (Figure 8).

### 3.6 Attitudes toward healthy nutrition and health-related quality of life

A comprehensive CHAID analysis revealed a significant (*p* < 0.001, *F* = 16.8, df1 = 1, df2 = 4063) impact of attitudes toward healthy nutrition on HRQoL (EQ-Index). The decision tree categorized patients into two groups:

1. Strongly or mostly agree: EQ-Index = 0.862 ± 0.198 SE
2. Neutral, undecided, mostly or strongly disagree: EQ-Index = 0.835 ± 0.221 SE

No significant differences were found between the “neutral or undecided” and “mostly or strongly disagree” groups. Similar results were obtained using the Kruskal-Wallis test. Table 2 presents the corresponding pairwise comparisons of attitudes toward healthy nutrition, along with a visual representation.

**Table 2:** EQ-5D-5L score: pairwise comparisons of the attitude toward healthy nutrition (Kruskal-Wallis Test).

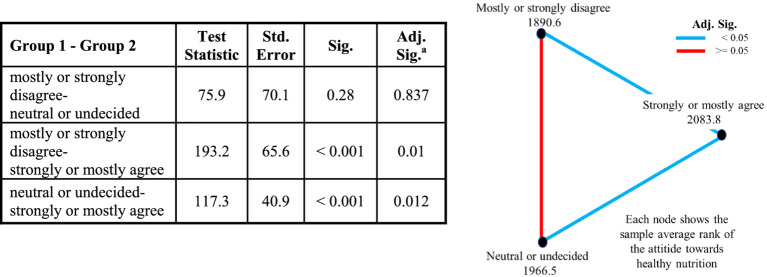

Each row tests the null hypothesis that the Group 1 and Group 2 distributions are the same. Asymptotic significances (2-sided tests) are displayed. The significance level is 0.050. ^a^Significance values have been adjusted by the Bonferroni correction for multiple tests.

When age was added to the model and analyzed using Quade’s nonparametric ANCOVA (*F* = 8.4, DFH = 2, DFE = 4,062, p < 0.001), the adjusted significance levels for the three pairwise comparisons remained similar to those shown in Table 2 (*p* = 0.289, *p* = 0.001, *p* = 0.002).

### 3.7 Characterization of the participants who strongly or mostly agree with healthy nutrition by age, sex, education and net monthly household income

We aimed to characterize participants who strongly or mostly agreed with healthy nutrition based on age, sex, education, and net monthly household income.

**Figure 9:**
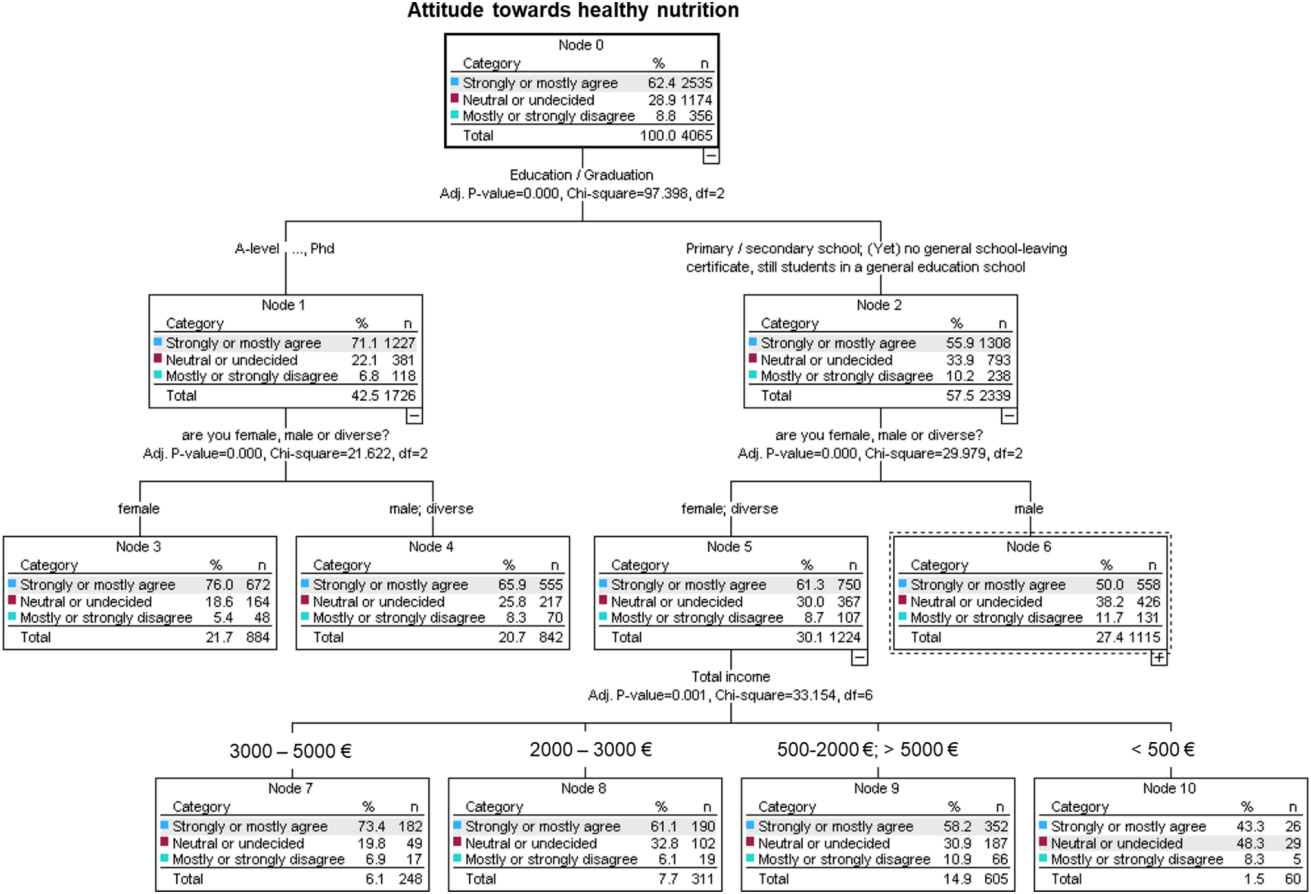
Exhausted chi-squared automatic interaction detection decision tree of various risk factors (age, sex, education, and net monthly household income) for attitude toward healthy nutrition.

A comprehensive CHAID analysis (Figure 9) with 10-fold cross-validation achieved a 96.9% correct classification rate for participants who strongly or mostly agreed with healthy nutrition. Education emerged as the most significant prognostic variable, followed by sex and net monthly household income. Age did not significantly influence the classification in this model. The maximum tree depth was three, resulting in 13 classification rules. The three classification rules with the highest probabilities of strongly or mostly agreeing with healthy nutrition are:

- Female with tertiary education (colleges, universities, professional schools): 76.0% probability of strongly or mostly agreeing with healthy nutrition.
- Female or diverse with primary or secondary education and a net monthly income of €3,000–€5,000: 73.4% probability.
- Male or diverse with tertiary education: 66.0% probability.

## 4 Discussion

An online-representative cross-sectional survey examined the association between socio-demographic factors and individuals’ perspectives on nutrition. Two-thirds of respondents considered nutrition important. Factors associated with prioritizing healthy nutrition include a higher education level, female sex, higher household income, a good health status, older age and congruence with certain social milieus as defined by Sinus.

The CHAID analyses show that education was the strongest predictor of attitudes toward healthy nutrition, followed by sex and income. Notably, females with tertiary education and higher household income were most likely to prioritize healthy nutrition. In contrast, individuals with lower income or education levels demonstrated reduced engagement with nutrition, reinforcing the importance of targeted public health initiatives to bridge these disparities. Furthermore, participants with a positive attitude toward healthy nutrition exhibited significantly higher HRQoL scores, as measured by the EQ-5D-5L Index.

When asked about the main reasons for their eating habits, participants identified taste preferences as the primary factor influencing food choices, followed by cost and health considerations. The diets of the participants revealed that 10.5% did not consume meat, 28.6% identified as flexitarians, and 54.1% as omnivores, with women demonstrating a stronger preference for plant-based diets.

Another interesting finding from the study was the high proportion of respondents indicating the motivation to adopt a more plant-based diet subsequent to recommendations from physicians or scientists.

### 4.1 Comparison with previous research

A survey commissioned by the German Federal Ministry of Food and Agriculture (BMEL) in 2023, involving 1,001 participants, yielded comparable findings with regard to the prevalence of plant-based diets in Germany. In particular, the presented data on the German population regarding vegans (1,5% in this survey; 2% in the BMEL survey) and vegetarians (10.5% of our participants and 10.0% in the BMEL refraining from meat) (3). This also aligns with recent data from the Institut für Demoskopie (IfD) Allensbach, showing that 8.12 million people in Germany identify as vegetarians and 1.52 million as vegans (32, 33), which would translate into approximately 9.59% vegetarians and 1.8% vegans in the German population (84.7 million in 2023) (34). In contrast, earlier data from the NVS II (Nationale Verzehrsstudie II), collected between 2005 and 2007 from nearly 20,000 German citizens aged 14 to 80, showed a remarkable difference, with only 0.1% classified as vegans and 1.6% as vegetarians (35). The “Study on the Health of Adults in Germany” (German: Studie zur Gesundheit Erwachsener in Deutschland - DEGS1), conducted from 2008 to 2011 indicated a gender distribution of vegetarians of 6.1% among females and 2.5% among males, while in the BMEL survey of 2023 12 % of the female participants followed a vegetarian diet, compared to 6 % of men (3). In our data, the majority of vegetarian or vegan-oriented participants were also female (69.4% of the vegetarians, 81.7% of the vegans, Figure 4). In line with previous research (36), taste preferences were the main factor influencing food choices according to our survey data. Cost and health also played significant roles. In comparison, the 2023 BMEL survey also found taste being the primary factor in food choices, followed by healthy nutrition (3). An individual’s personal network also appears to play a significant role in shaping eating practices (37, 38). Meat consumption is notably reduced when someone in the household follows a vegetarian diet, with a smaller impact observed if a person’s social circle includes a vegetarian friend or relative (39).

One fifth of the survey participants regularly bought organic foods, but most reported a combination of conventional and organic groceries when shopping. In recent years, the market-share of organic-farmed food in the total food sales in Germany has steadily increased and reached 7 % in 2021 (40). This growth reflects Germany’s market development. In 2021, Germany had a per capita spending of over 191 € on organic food (41).

### 4.2 Nutrition vs. education and social status

Education and higher income were closely associated with the importance attributed to nutrition in our survey, confirming earlier research showing that a lower social status was associated with poor eating habits (42). A 2013 study revealed that adults in Germany with lower education levels consume energy-dense foods more frequently and eat fewer fruits and vegetables compared to those with higher education levels (43). Similarly, a study encompassing Eastern, Central, and Western Europe found that higher education, occupational status, and fewer economic difficulties positively correlate with healthier food habits (44). Data from the DEGS 1 study (2008–2011) suggested that individuals with higher educational levels, city residents, and physically-active individuals (≥4 h of sports per week) were more likely to follow a vegetarian diet, which is linked to fewer energy-reduced drinks, beer, and wine, while increasing intake of tea, fruits, and vegetables (22). Another study suggests that targeting awareness of food sustainability to specific segments of the population is essential, as certain socio-demographic characteristics appear to be associated with a lack of awareness about the sustainability of their diet (45). It is important to point out that due to the different employed methodologies; a direct comparison is not possible.

### 4.3 Role of medical doctors and nutrition in medical education

Advice from health professionals appears to be one of the strongest drivers of dietary changes, even among resistant groups, contributing to reduced health disparities and the promotion of environmentally sustainable practices (46–48). The willingness of nearly one-third of participants in our study to consider dietary changes based on recommendations of medical doctors or researchers underlines the crucial role that physicians play in guiding patients toward healthier nutrition. However, nutrition remains underrepresented in German medical education (49), leading to inadequate levels of knowledge among students. Our findings highlight the need for an enhanced focus on nutrition in the medical curriculum, ensuring that future physicians are well-equipped to provide comprehensive nutritional advice and support to their patients (50, 51).

The Physicians Association for Nutrition (PAN) advocates the integration of more nutritional education in medical training. The “Eat this” (German: “Iss Das!”) webinar series, organized by PAN University Groups, has reached thousands of medical students and has been recognized as an elective subject at several universities, including Cologne and Munich (LMU) (52). Additionally, Charité – Universitätsmedizin Berlin, one of Germany’s largest medical faculties, has introduced an innovative elective course focused intensively on nutrition, fasting, and planetary health. This course aims to profoundly acquaint medical students with the significance of nutrition in health and disease prevention. The successful implementation at Charité may serve as a model for expanding nutritional education in medical curricula nationwide, addressing the educational gap in this field (53).

These findings call for enhanced public health strategies to promote diverse, nutrient-rich diets and point to the necessity of integrating nutritional education more comprehensively into medical training and patient care as well as the provision of more sustainable and healthier food in hospitals and other public institutions (54, 55).

### 4.4 Strengths and limitations

The study employed an online access panel for survey administration, chosen for its rigorous standards in participant selection and maintenance, along with the implementation of a quota system (56). This method was selected to ensure that insights into the utilization and acceptance of TCIM in the German population were representative of the broader population. The online mode was specifically chosen due to the sensitivity of personal health-related questions. The use of an access panel resulted in the exclusion of specific parts of the population, such as those without online access or with low online affinity such as the very elderly due to their lower likelihood of internet access.

Moreover, there is a potential for self-selection bias, as individuals with a greater interest in health and nutrition may have been more likely to participate in the study. Those not interested in such themes might have declined to participate, leading to an overrepresentation of health-conscious individuals. This could influence the results, skewing them toward more favorable attitudes and behaviors related to health and nutrition than would be present in the general population.

Through its cross-sectional design the study yielded primarily descriptive data. It is important to acknowledge that cross-sectional designs are not suitable for determining causal relationships between variables. Additionally, dietary data were collected using self-report methods, which, while practical for gathering information, are prone to various biases and inaccuracies.

The study leverages decision trees as part of its analytical approach, reflecting the common format of decision trees or rule-based systems in medical guidelines, with which physicians are typically familiar. This methodology aligns with medical practices, facilitating the interpretation and application of findings in clinical settings.

Due to a relatively low response rate of 21.5% the study encountered further limitations, raising concerns about the extent to which the results can be generalized to the broader population. To mitigate this issue and enhance the generalizability of the findings, the data were weighted to account for factors such as gender, age, education, federal state, and city size.

These limitations highlight the need for focused research to fully understand the role, efficacy, and impact of specific factors influencing food choices. For a more precise assessment of the effects of incentives, experimental studies that track actual behavioral changes are essential (57, 58).

### 4.5 Future research

Future transdisciplinary research between nutrition science, public health, behavioral science as well as food or agricultural and environmental science, leveraging high-quality study designs and methods, is essential to establish causal relationships between influential factors and food choices and to gain deeper insights into and further explore behavioral shifts toward sustainable and healthy nutrition, aiming to benefit both individual health and the planet. Randomized clinical trials could investigate the efficacy of, e.g., gender-or milieu-adapted nutrition programs on long-term health outcomes, explore the influence of cultural or religious factors on food choices, and assess the impact of nutrition-trained physicians in patient settings in regard to optimizing or shifting dietary patterns. Especially programs making inroads to milieus and income-groups with lower interest in nutrition with the help of intervention mapping designs (59), might help tackle health challenges more effectively on a population level. Further studies should look at most effective strategies for promoting healthy and sustainable nutrition at the individual, community, and policy levels, considering cultural, economic, and environmental factors.

## 5 Conclusion

Our survey portrays numerous associations between socio-demographic factors and selected dietary habits. It may serve as a future basis for targeted interventions and policies to promote healthier eating habits and to address the diverse needs of different sociodemographic groups. Compared to former surveys, the adoption of flexitarian diets by a growing proportion of the German population suggests a shift toward healthier, more sustainable eating habits. The transformation toward more plant-based diets holds potential for improving both personal and planetary health.

## Data Availability

The raw data supporting the conclusions of this article will be made available by the authors, without undue reservation.

## In memoriam

The authors dedicate this article to Boike Rehbein (†11.6.2022). Boike Rehbein was involved in the planning and initiation of the project and sadly passed away during the project.
