# Supplementary material for: Attitudes toward healthy nutrition in Germany — results from an online-representative cross-sectional survey
Source: Front Nutr. 2025 Jan 3;11:1480980. doi: 10.3389/fnut.2024.1480980 (PMC11783846; doi:10.3389/fnut.2024.1480980)
Supplement: Supplementary file 1 [file Table_1.docx]

Supplementary Material

**1 Supplementary Figures and Tables**

**1.1 Figures**

**Supplementary Figure 1A, 1B and 1C** “Healthy nutrition is an important topic in my life” - depending on attitude towards TCIM, gender, total income, high-school diploma, age groups, health status and Sinus Main Milieus**®** (all branches of the decision trees are significant).


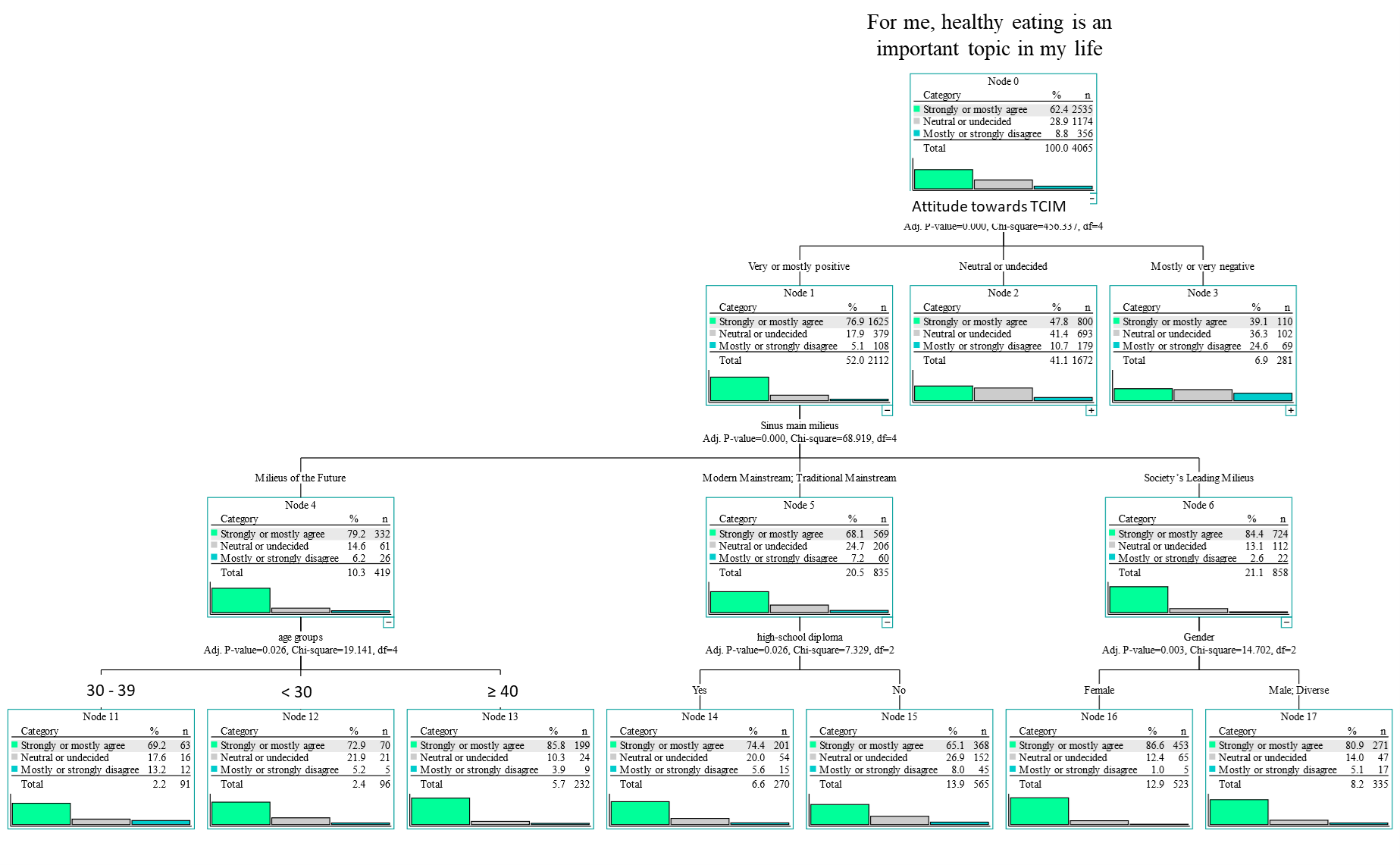


**Supplementary Figure 1A**


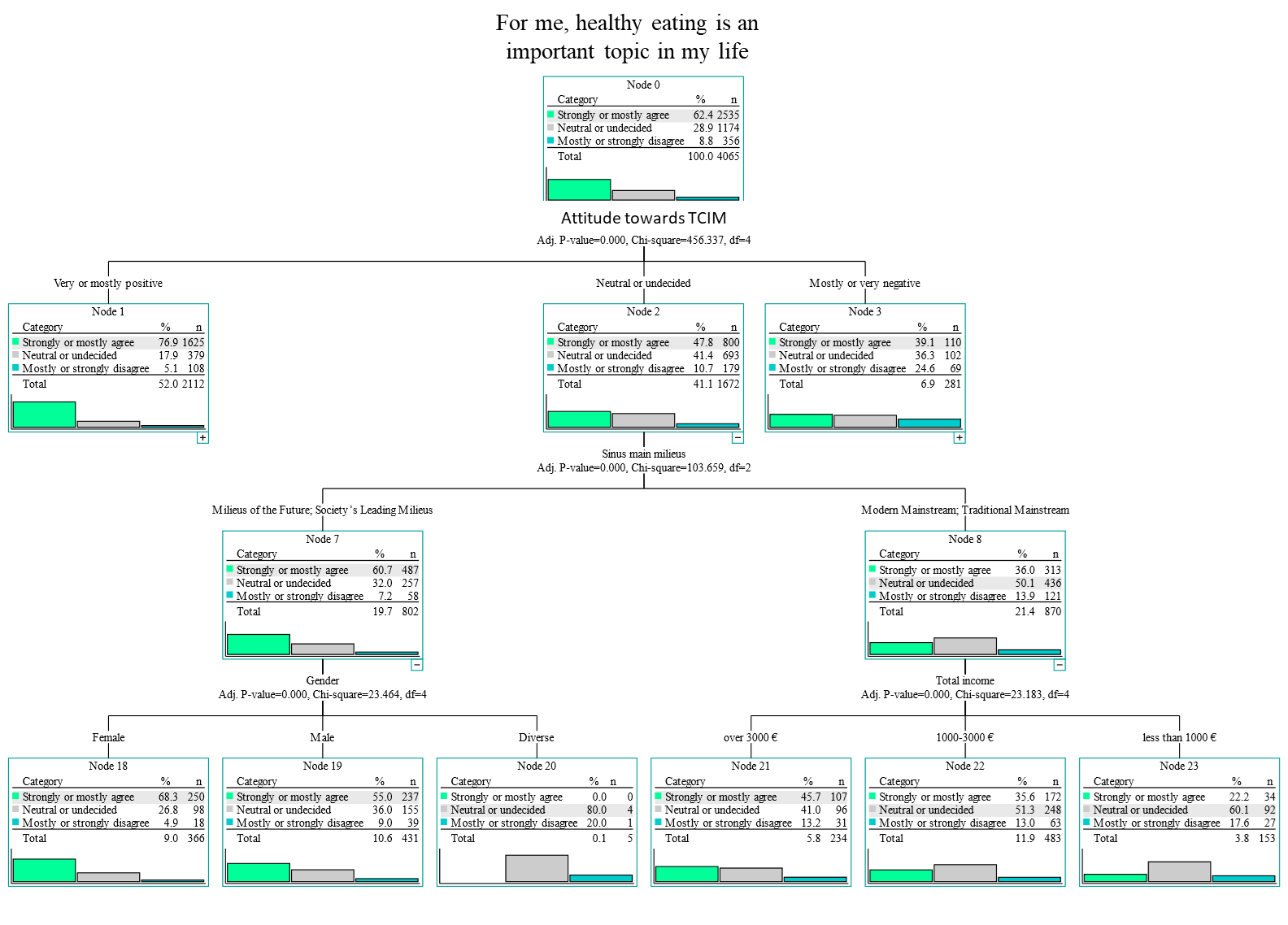


**Supplementary Figure 1B**


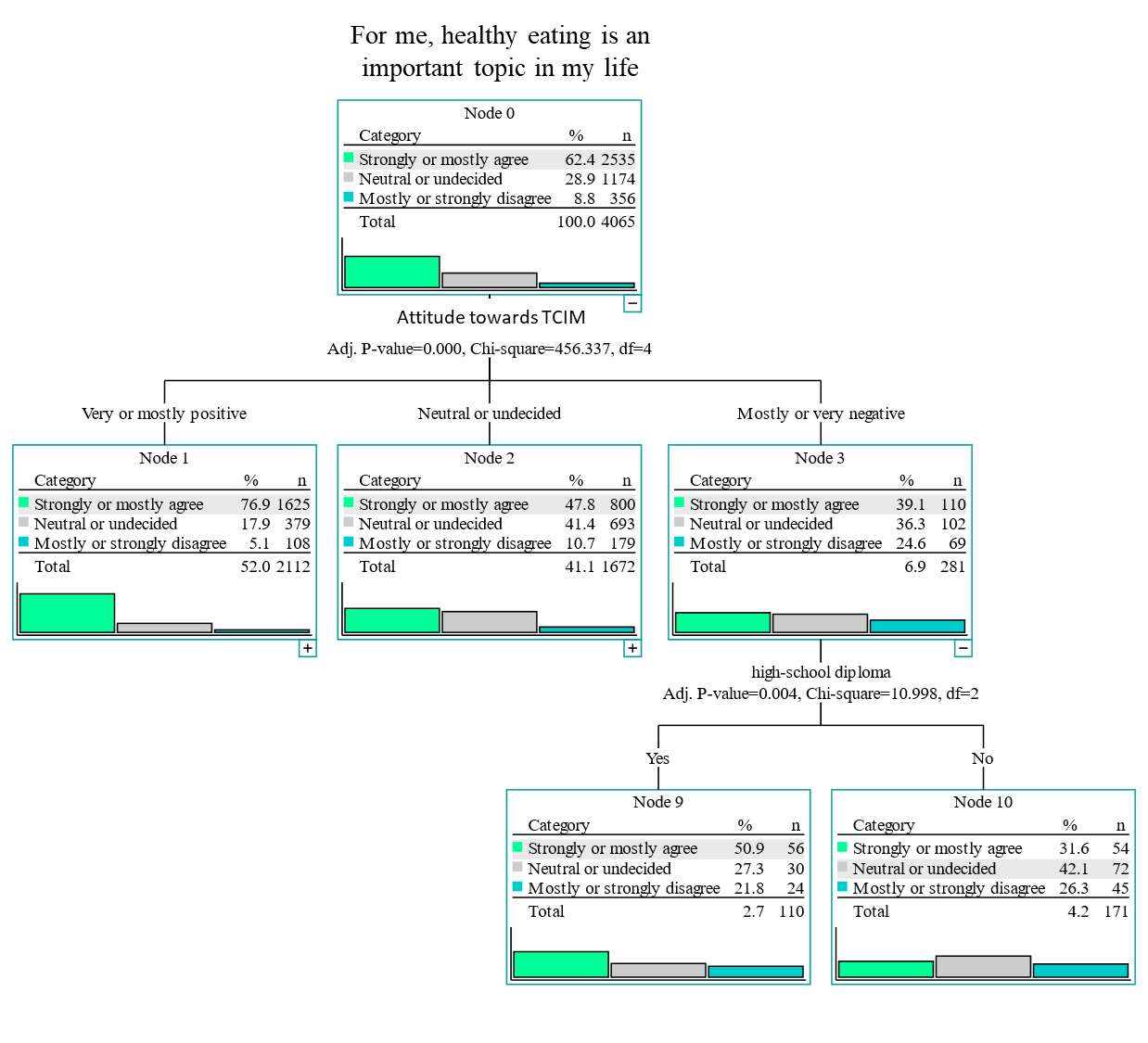


**Supplementary Figure 1C**


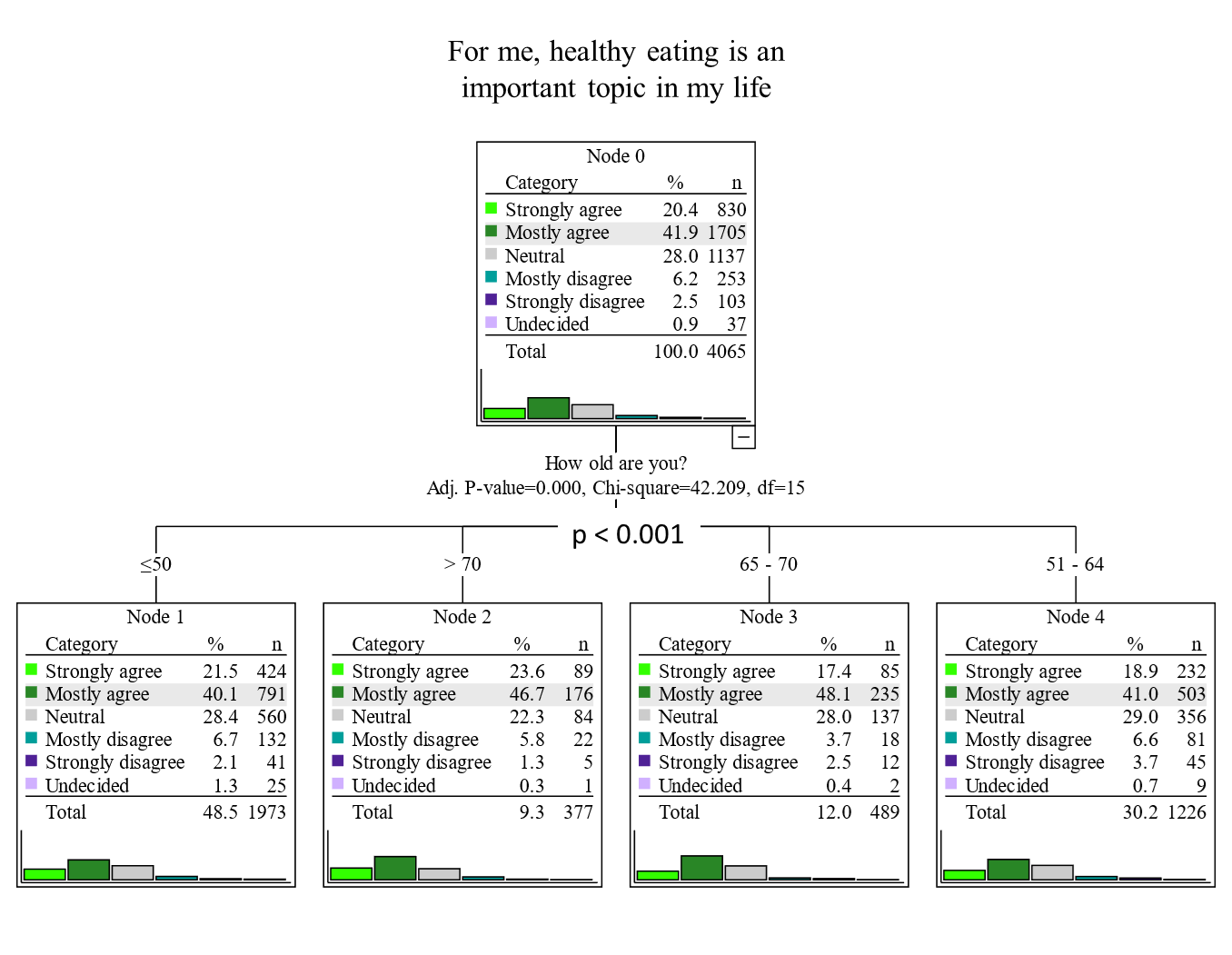


**Supplementary Figure 2**: Healthy nutrition is an important topic in my life – Age


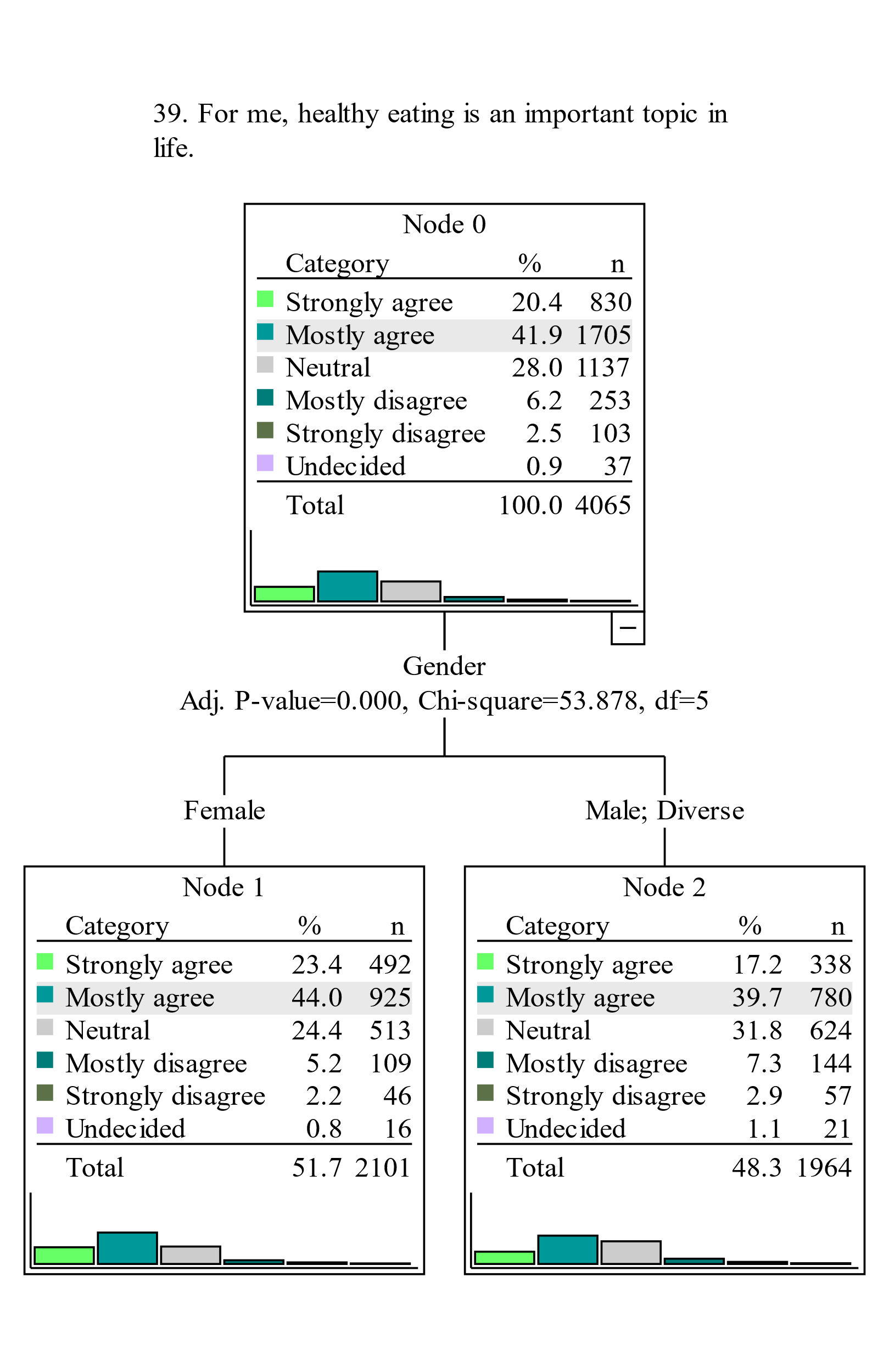


39. For me, healthy eating is an important topic in

life.

Node 0

Category

%

n

20

.4

830

Strongly agree

41

.9

1705

Mostly agree

28

.0

1137

Neutral

6

.2

253

Mostly disagree

2

.5

103

Strongly disagree

0

.9

37

Undecided

Total

100

.0

4065

Gender

**Supplementary Figure 1:** For me, healthy eating is an important topic i

Adj. P-value=0.000, Chi-square=53.878, df=5

Female

Male; Diverse

Node 1

Node 2

Category

%

n

Category

%

n

23

.4

492

Strongly agree

17

.2

338

Strongly agree

44

.0

925

Mostly agree

24

.4

513

Neutral

39

.7

780

Mostly agree

31

.8

624

Neutral

5

.2

109

Mostly disagree

7

.3

144

Mostly disagree

2

.2

46

Strongly disagree

0

.8

16

Undecided

2

.9

57

Strongly disagree

1

.1

21

Undecided

Total

51

.7

2101

Total

48

.3

1964

**Supplementary Figure 3** Healthy nutrition is an important topic in my life. There are significant (p < 0.001)) differences between female and men/diverse but not between men and diverse (p = 0.936).

**1.2 Tables**

**Supplementary Table 1**: **Questionnaire – translated questions on nutrition**

1. Which of the following answers applies to you? How do you eat?

| O | Omnivorous |
| --- | --- |
| O | Omnivorous, but with few animal products [flexitarian] |
| O | Plant-based - almost without animal products (max. 5%) |
| O | Vegetarian, including dairy products and eggs [lacto-ovo-vegetarian] |
| O | Vegetarian plus fish [pescetarian] |
| O | Vegan, without animal products |
| O | Raw food |
| O | Other [please specify] |
| O | Don't know |

1. Which answer option applies to you? Healthy nutrition is an important topic in my life.

| O | Strongly agree |
| --- | --- |
| O | Mostly agree |
| O | Neutral |
| O | Mostly disagree |
| O | Strongly disagree |
| O | Undecided |

1. Which answer options apply to you? My 3 main reasons for the way I eat are: [You can select up to 3 options]

| O | Personal taste preferences |
| --- | --- |
| O | Family traditions / habit |
| O | Cultural traditions |
| O | Health aspects |
| O | Costs |
| O | Ethical aspects |
| O | Practicality/convenience (fast, simple, available) |
| O | Compatibility with daily routine/family/partner/roommates |
| O | Other: [please specify] |

1. Which answer option applies to you? I buy organic food...

| O | ...exclusively. |
| --- | --- |
| O | ...predominantly. |
| O | ...also. |
| O | ...sometimes. |
| O | ...never. |

1. Which option applies to you? If doctors or scientists recommended abstaining from foods of animal origin, I would...

| O | ... definitely do it. |
| --- | --- |
| O | ...seriously consider this. |
| O | ...maybe think about it. |
| O | ...rather not do it. |
| O | ...certainly not do it. |

1. What actions are you currently taking in your diet that you consciously do for health reasons? (Multiple answers possible)

| O | I eat fewer animal products |
| --- | --- |
| O | I only eat certain foods of animal origin (certain types of meat, fish, etc.) |
| O | I practice intermittent fasting or other forms of fasting |
| O | I consciously leave breaks between meals |
| O | I eat less sugar and sweets |
| O | I eat less fast food and junk food |
| O | I prepare food myself and freshly |
| O | I eat whole grain products |
| O | I eat lots of vegetables and fresh food |
| O | I abstain from alcohol |
| O | I eat low-fat |
| O | I eat low-carb |
| O | Other [please specify] |

1. Which answer option applies to you? If my doctor recommended me to fast for health reasons, I would...

| O | ...definitely do it. |
| --- | --- |
| O | ...seriously consider it. |
| O | ...maybe think about it. |
| O | ...rather not do it. |
| O | ...certainly not do it. |

1. Which answer option applies to you? Would you eat a more plant-based diet if, ...

|  | Strongly agree | Mostly agree | Mostly disagree | Strongly disagree | Don't know |
| --- | --- | --- | --- | --- | --- |
| your doctor recommended it? | O | O | O | O | O |
| science and politics recommended it for society as a whole? | O | O | O | O | O |
| more delicious plant-based dishes were offered in the canteen/cafeteria at your workplace? | O | O | O | O | O |
| more people in your surroundings were eating a plant-based diet? | O | O | O | O | O |
| high quality plant-based food products were cheaper? | O | O | O | O | O |
| a plant-based way of eating was more widespread in society? | O | O | O | O | O |
| there were better options available in supermarkets and restaurants? | O | O | O | O | O |
| Plant-based foods tasted better? | O | O | O | O | O |
